# Chronic Intestinal Stasis†Abstract of paper, with lantern slide demonstration, presented before the Cumberland Medical Society, April 19, 1913.

**Published:** 1914-01

**Authors:** William Seaman Bainbridge

**Affiliations:** Professor of Surgery, N. Y. Polyclinic Medical School and Hospital


### Chronic Intestinal Stasis.†

BY WILLIAM SEAMAN BAINBRIDGE, A. M., SC. D., M. D.
Professor of Surgery, N. Y. Polyclinic Medical School and Hospital.

The work of W. Arbuthnot Lane,* of London, has opened a distinctly new line of investigation with regard to a large class of maladies associated with impaired function of some part of the

†Abstract of paper, with lantern slide demonstration, presented before the Cumberland Medical Society, April 19, 1913.

*In June, 1913, for his great contribution to surgery, Mr. Lane was created a Baronet.

alimentary canal and of organs closely related thereto. General auto-introxication, chronic constipation, visceroptosis, floating kidney, gallstones, duodenal ulcer, mucous colitis, pericolitis, chronic appendicitis, and other conditions traceable to perverted function of these parts of the organism, must now be studied from a different point of view from that which has been brought to bear in the past. Lane has pressed forward in the effort to discover first causes, and in doing so he has demonstrated clearly that in health maintenance the question of prime importance is body-drainage—the non-absorption of poisons, and the elimination of whatever poisonous matter may be produced within the alimentary canal, before there has been inaugurated a vicious cycle of events which may be the forerunner of disastrous end-results. Attention has thus been focused more directly upon the previously obscure phenomena of intestinal stasis, and a plausible explanation has been offered of the causes underlying the evolution of these phenomena.

Lane has demonstrated to his own satisfactioon and to that of a large number of surgeons, in Europe and America, that intestinal stasis, as he calls this slowing of the drainage current, is the result of the abnormal fixation of certain portions of the canal, with a dropping of the tube on either side of the fixed point, thus producing a kink. The kinking of the intestine prevents the free passage of its contents, causes a "puddling" in the dependent portions, a damming back and infection of the material and a general slowing of the drainage. His definition of this state of affairs, this "chronic intestinal stasis," is: "Such an abnormal delay in the passage of the intestinal contents through a portion or portions of the gastro-intestinal tract as results in the absorption of a greater quantity of toxic or poisonous materials than can be treated effectually by the organs whose function it is to convert them into products as innocuous as possible to the tissues of the body." This reabsorption and auto-intoxication, according to Lane, leads to a general lowering of the resistance of the body and to the concomitant increase in susceptibility to various diseases. This increased susceptibility, in his opinion, finds an expression in various conditions to which he has applied the term "end-results." His view, it will be noted, is thus the direct antithesis of that of many other observers, who consider such diseases as causative factors in the production of chronic intestinal stasis. Notable examples of what Lane has diagnosed as "end-results" are: cancer of the stomach, intestine, biliary ducts, or pancreas; visceral tuberculosis; rheumatoid arthritis.

The explanation offered by Lane for the conditions which lead to chronic intestinal stasis is a purely evolutionary one, as opposed to the hereditary, congenital and toxic theories held by others who have made a study of intestinal obstruction in general. According to Lane, the toxic symptoms are secondary; others hold them to be primary or causative of the mechanical changes which produce stasis.

The ptosis of the abdominal viscera, according to Lane's theory, is, broadly speaking, the result of the assumption, by man, of the upright position. In early life, this ptosis is the result of an abnormal distention of the intestine, consequent upon too frequent feeding, or upon the continued use of an unsuitable diet. In later years it is brought about or accentuated by the erect posture which man assumes during the waking hours. The resulting pressure and strain may produce distinct changes, which may take the form of *evolutionary bands,* as Lane calls them. These bands are practically without blood supply, and are not to be confounded with *inflammatory adhesions,* which are apt to have a generous blood supply. These evolutionary bands exist primarily for the advantage of the individual, for the purpose of supporting the intestines and preventing them from dropping downward. These bands do not develop with equal strength throughout, unfortunately, and as a consequence the bowel is held up firmly in some points and allowed to sag in others, the result being the angulation, or kink, at the point of support.

The formation of these evolutionary bands leads to kinking of the intestine at certain points of predilection, as demonstrated by Lane: (1) pylorus; (2) duodeno-jejunal junction; (3) different points along the terminal coil of the ileum; (4) appendix; (5) hepatic flexure; (6) splenic flexure; (7) sigmoid flexure; (8) rectum.

The symptoms which result from chronic intestinal stasis vary in different individuals, with the degree of obstruction, and with the concomitant "end-result" manifestations. They may be briefly catalogued as follows: (1) Headache, severe and frequent; (2) nausea, followed by retching or vomiting; (3) anorexia, almost constantly present; (4) loss of weight; (5) coldness of extremities; (6) mental apathy; (7) constipation; (8) foul taste in mouth, often accompanied by foul breath, carious teeth, and furred tongue; (9) abdominal distention, relieved by eructation, the passage of flatus, or an action of the bowels; (10) abdominal tenderness over the area of fixation; (11) skin-staining; (12) breast changes, simulating chronic mastitis, in the early stages, and cystic degeneration in the later stages; (13) general muscular pain and more or less marked stiffness of joints.

Stasis cases have been classified, according to predominant symptoms, under the following types:

(1) *Obstructive.* These have usually been regarded as having duodenal ulcer, cancer of the stomach, or "nervous dyspepsia."

(2) *Toxic,* in which the symptoms range from occasional "bilious attacks," through the various types of "indigestion," atonic constipation," to neuroses of divers kinds.

(3) *Mixed Type,* presenting symptoms of the obstructive and toxic types.

(4) *End-result Type,* comprising such affections as intestinal cancer, gall-stones, tuberculosis, rheumatoid arthritis, Still's disease, etc.

The diagnosis of chronic intestinal stasis is made by the clinical symptoms, plus careful radiographic study, the degree of obstruction and the location of the obstructing kink being determined by the rapidity of the passage through the intestine of bismuth.

The treatment depends upon the degree of stasis. In atonic or asthenic individuals, where there is general loss of muscular tone and nervous energy, with only slight degrees of ptosis of the hollow viscera, complete cure may be effected by means of abdominal supports, tonic regime, building up the nutrition, and rest, until nerves and muscles have regained their normal tonus. Liquid paraffin (oleum mineralis Russicum) given in from ½ to 1 ounce doses two or three times a day on an empty stomach has been found particularly helpful in this class of cases.

It is held by Lane and his followers that in the vast majority of instances, intelligent management by the internist will prevent chronic intestinal stasis, and that the milder degrees, promptly recognized and carefully treated by the measures outlined, may be cured without recourse to surgical measures. Once allowed to progress, however, to the more marked degrees of stasis, with definite kinking, and with clearly characteristic symptoms, surgery must be brought into requisition. The extent of the operative interference must be contingent upon conditions found upon laparotomy. In some cases, the cutting of the evolutionary bands, and the restoration of the angulated or misplaced hollow viscera or other organs, to their normal position, will effect the return of normal function and the disappearance of the symptoms. In other cases, particularly where broad bands are formed, with adhensions between different portions of the gut, with possible involvement of other organs, such as ovaries, gall-bladder, liver, etc., it is necessary to resort to ileocolostomy (Lane's "short-circuiting" operation), or colectomy.

